# Resistance to Mucosal Lysozyme Compensates for the Fitness Deficit of Peptidoglycan Modifications by *Streptococcus pneumoniae*

**DOI:** 10.1371/journal.ppat.1000241

**Published:** 2008-12-12

**Authors:** Kimberly M. Davis, Henry T. Akinbi, Alistair J. Standish, Jeffrey N. Weiser

**Affiliations:** 1 Department of Microbiology, University of Pennsylvania School of Medicine, Philadelphia, Pennsylvania, United States of America; 2 Divisions of Pulmonary Biology and Neonatology, Cincinnati Children's Hospital Medical Center, Cincinnati, Ohio, United States of America; Schepens Eye Research Institute, United States of America

## Abstract

The abundance of lysozyme on mucosal surfaces suggests that successful colonizers must be able to evade its antimicrobial effects. Lysozyme has a muramidase activity that hydrolyzes bacterial peptidoglycan and a non-muramidase activity attributable to its function as a cationic antimicrobial peptide. Two enzymes (PgdA, a *N*-acetylglucosamine deacetylase, and Adr, an *O*-acetyl transferase) that modify different sites on the peptidoglycan of *Streptococcus pneumoniae* have been implicated in its resistance to lysozyme *in vitro*. Here we show that the antimicrobial effect of human lysozyme is due to its muramidase activity and that both peptidoglycan modifications are required for full resistance by pneumococci. To examine the contribution of lysozyme and peptidoglycan modifications during colonization of the upper respiratory tract, competition experiments were performed with wild-type and *pgdAadr* mutant pneumococci in lysozyme M-sufficient (LysM^+/+^) and -deficient (LysM^−/−^) mice. The wild-type strain out-competed the double mutant in LysM^+/+^, but not LysM^−/−^ mice, indicating the importance of resistance to the muramidase activity of lysozyme during mucosal colonization. In contrast, strains containing single mutations in either *pgdA* or *adr* prevailed over the wild-type strain in both LysM^+/+^ and LysM^−/−^ mice. Our findings demonstrate that individual peptidoglycan modifications diminish fitness during colonization. The competitive advantage of wild-type pneumococci in LysM^+/+^ but not LysM^−/−^ mice suggests that the combination of peptidoglycan modifications reduces overall fitness, but that this is outweighed by the benefits of resistance to the peptidoglycan degrading activity of lysozyme.

## Author Summary

For many successful pathogens, their surfaces must be able to adapt to different host environments, or to avoid host immune components, to establish infection. Bacterial pathogens, for example, are known to modify their cell walls, which are comprised largely of peptidoglycan. Our study focuses on peptidoglycan modifications by *Streptococcus pneumoniae*, which initiates interaction with its host by colonizing the mucosal surface of the upper respiratory tract. Two proteins (PgdA and Adr) that modify the cell wall of *S. pneumoniae* have each been associated with resistance to lysozyme, which cleaves peptidoglycan and is one of the most abundant antimicrobial factors in the human respiratory tract. Using defined bacterial mutants together with mice that express or lack lysozyme, we show that the full resistance to lysozyme requires modifications by both proteins. These cell wall modifications each come at a significant fitness cost to the bacterium. This fitness cost, however, is outweighed by the benefits of lysozyme resistance *in vivo*. Our study, therefore, demonstrates the relationship between a bacterial pathogen and a host defense mechanism that imparts a substantial selective pressure on organisms that colonize the mucosal surface.

## Introduction

For many pathogens, colonization of mucosal surfaces is a prerequisite for events leading to disease. At these sites the host elaborates numerous antimicrobial factors that may reduce the burden of colonizing organisms. Lysozyme, a prominent member of these antimicrobials, is found in high concentrations (>500 µg/ml) in mucosal surface fluids such as those lining the upper respiratory tract [1]. Lysozyme is expressed by the epithelia and is also a major component of the granules of neutrophils, which may be recruited when the mucosa is acutely inflamed [2],[3],[4]. Lysozyme has two distinct antibacterial activities [5],[6]. Its enzymatic muramidase activity hydrolyzes the conserved ß-1,4 glycosidic bond between *N*-acetyl glucosamine (GlcNAc) and *N*-acetyl muramic acid (MurNAc), the disaccharide residues of the peptidoglycan backbone. Hydrolysis of the glycan strands leads to degradation of the cell wall and bacterial lysis. In addition, an antibacterial activity is observed with catalytically inactive lysozyme [6]. This non-muramidase activity has been attributed to the disruption of bacterial membrane function by an inherent nine amino acid cationic antimicrobial peptide (CAMP) [5],[7].

The contribution of lysozyme to innate host defense has been analyzed in genetically- modified mice. Over-expression of rat lysozyme in the lungs of transgenic mice was associated with enhanced killing of group B streptococci and *Pseudomonas aeruginosa* [8]. Mice have two different lysozyme genes, *LysM*, expressed by myeloid and epithelial cells and *LysP*, expressed by Paneth cells in the gut. LysM^−/−^ mice show defective clearance of *P. aeruginosa* [9] and *Klebsiella pneumoniae* from their lower airways [3]. While these studies demonstrate a protective role of lysozyme for opportunistic pathogens in normally sterile sites, its impact on the mucosal flora is unknown.

Another consideration is that bacteria modify the glycan backbone of their peptidoglycan, and these modifications may affect their sensitivity to lysozyme [10],[11],[12],[13],[14],[15],[16],[17],[18],[19]. In *Staphylococcus aureus*, for example, *O*-acetylation at position C-6 on MurNAc and d-alanine esters on its cell wall-linked teichoic acid have an additive effect on resistance to muramidase and non-muramidase activities of lysozyme *in vitro* [13]. Organisms that reside on mucosal surfaces where lysozyme is particularly abundant must have the ability to evade its antibacterial effects. However, the contribution of cell wall modifications, which affect lysozyme resistance, to bacterial survival on mucosal surfaces has not been examined.

*Streptococcus pneumoniae* (the pneumococcus) is a leading extracellular Gram-positive pathogen that commonly colonizes the human nasopharynx. Although colonization is generally asymptomatic, infection induces an acute inflammatory response characterized by a brisk influx of neutrophils. Because the organism is coated by capsular polysaccharide (CPS) covalently attached to its cell wall [20], it is capable of evading phagocytic clearance and causing invasive disease. Two enzymes have been associated with lysozyme resistance in *S. pneumoniae*: peptidoglycan *N*-acetylglucosamine deacetylase (PgdA) [10] and attenuator of drug resistance (Adr), which is an *O*-acetyltransferase [21]. These proteins modify a portion of the glycan residues; PgdA deacetylates GlcNAc (40–80% of GlcNAc residues, ∼10% of MurNAc residues) and Adr acetylates MurNAc [10],[21],[22]. Previous studies have shown that mutations in either of these genes are sufficient to increase sensitivity to chicken egg lysozyme *in vitro* [10],[21],[22]. Systemic infection of mice with a *pgdA* strain was associated with decreased virulence, although the role of lysozyme resistance in this effect was not established [22].

In this study, we determine the contribution of lysozyme to bacterial colonization using a murine model of carriage by *S. pneumoniae*. Our findings demonstrate the critical role of lysozyme, and peptidoglycan modifications that affect resistance to its muramidase activity, in dictating the composition of the microflora of the mucosal surface of the upper airway.

## Results

### Peptidoglycan modifications affect sensitivity to human lysozyme

**Figure 1:**
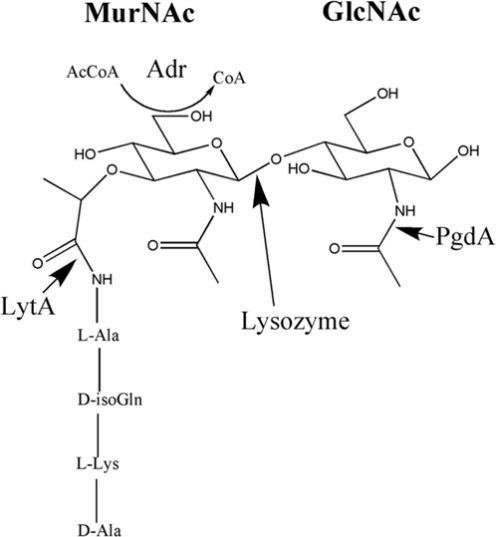
Predicted peptidoglycan structure. PgdA, an *N*-acetyl glucosamine, and Adr, an *O*-acetyltransferase, modify the MurNAc-GlcNAc disaccharide structure at the indicated sites. The major pneumococcal autolysin, LytA, cleaves the stem peptide attached to MurNAc. Lysozyme hydrolyzes the glycosidic bond between MurNAc and GlcNAc as shown.

**Figure 2:**
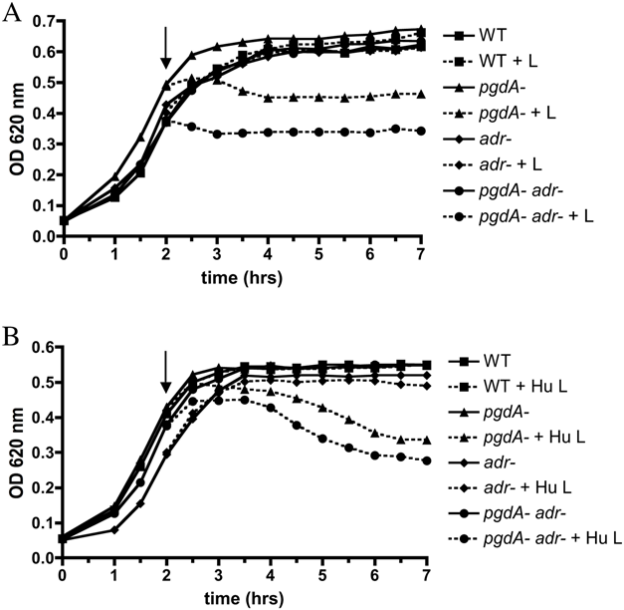
Effect of peptidoglycan modifying enzymes PgdA and Adr on growth of pneumococci in the presence or absence of lysozyme. Growth characteristics of the wild-type (WT) strain or defined mutants were compared by following the optical density (OD 620 nm). Once the broth culture reached mid-log phase, lysozyme (100 µg/ml) was added where indicated by an arrow. A) chicken egg lysozyme (+L) or B) recombinant human lysozyme (+Hu L). Graphs are representative of six independent experiments.

**Figure 3:**
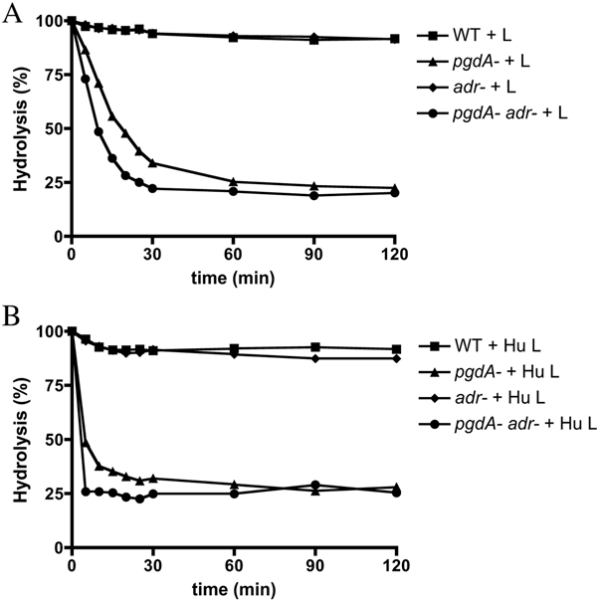
Effect of peptidoglycan modifying enzymes PgdA and Adr on hydrolysis of pneumococcal cell walls in the presence of lysozyme. Hydrolysis of peptidoglycan (50 µg/ml), purified from the wild-type (WT) strain or the defined mutants indicated with lysozyme (100 µg/ml) from A) chicken egg (+L) or B) human (+Hu L). Representative experiment showing percentage of hydrolysis based on the optical density (OD 600 nm) of each reaction at time 0 min.

To test the effects of peptidoglycan modifications, defined mutations were made in *pgdA* and *adr* in the pneumococcal strain TIGR4 based on the genomic information available for this isolate as described in the Methods. Figure 1 depicts the locations of *O*-acetylation (Adr) and *N*-deacetylation (PgdA) relative to the glycosidic linkage of MurNAc and GlcNAc that is hydrolyzed by lysozyme. The wild-type (WT), *pgdA*, *adr*, and *pgdAadr* strains showed equivalent growth characteristics in broth culture during log phase. Addition of chicken egg lysozyme (100 µg/ml) to mid-log phase cultures led to arrested growth for the *pgdA* mutant strains (*pgdA* and *pgdAadr*), but not the WT or *adr* strains (Fig. 2A). Growth arrest occurred more promptly for the *pgdAadr* than *pgdA* mutant suggesting a synergistic effect of the two genes on sensitivity to lysozyme. The results with the *pgdA* mutant were consistent with previous studies [10],[22], however the *adr* mutant of strain TIGR4 generated in this study did not have increased sensitivity to lysozyme as has been previously reported for a strain derived from R6 [21]. A similar pattern of sensitivity was observed using recombinant human lysozyme (100 µg/ml), although with the human enzyme bacterial lysis was more apparent (Fig. 2B). Purified insoluble peptidoglycan from each strain was tested to determine whether the effect of lysozyme on cultures correlated with its hydrolytic activity. For both chicken egg (Fig. 3A) and human lysozyme (Fig. 3B) only peptidoglycan from the mutants lacking *pgdA* were hydrolyzed. As with whole bacteria, peptidoglycan from the *pgdAadr* mutant showed greater sensitivity to lysozyme than the *pgdA* strain.

**Figure 4:**
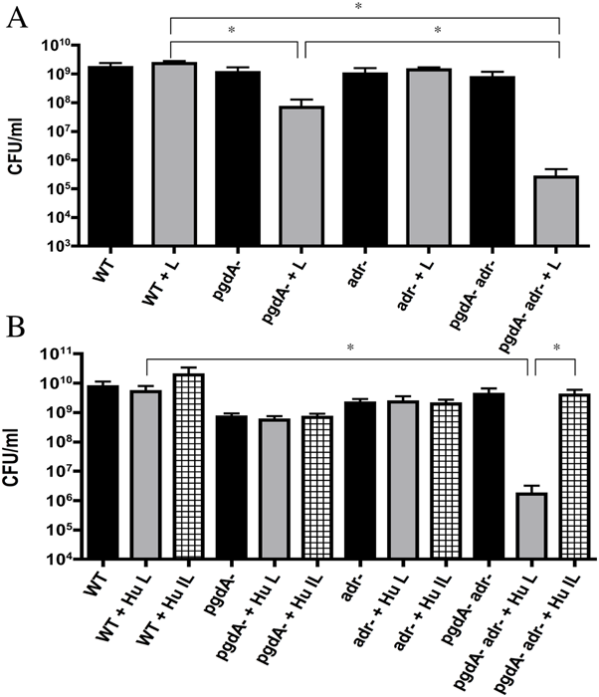
Effect of peptidoglycan modifying enzymes PgdA and Adr on viability of pneumococci in the presence of lysozyme. Once the broth culture of the wild-type (WT) strain or the defined mutants indicated reached mid-log phase, lysozyme (100 µg/ml) was added and viable counts (CFU/ml) were measured 5 hrs later. Strains tested were in a *lytA* background to eliminate effects of autolysis. Conditions included A) chicken egg lysozyme (+L) and B) recombinant human lysozyme (+Hu L) or heat inactivated recombinant human lysozyme (+Hu IL). Graphs are based on four independent determinations ±S.D. (^*^ p<0.05).

Pneumococcal growth is characterized by spontaneous autolysis in stationary phase because of the endogenous expression of its major amidase, LytA (Fig. 1). Since colonies of *pgdA* mutants generated in this study appeared more autolytic based on colony morphology, the *in vitro* effects of lysozyme were tested in a *lytA* background (Fig. 4). To determine the effect of lysozyme on pneumococcal survival, viable counts were measured five hours after addition of chicken egg (Fig. 4A) or human lysozyme (Fig. 4B) to mid-log phase cultures. The WT strain and *adr* mutant showed no loss of viability. Survival of both *pgdA* mutants was significantly reduced by treatment with chicken egg lysozyme, with a more significant effect on the *pgdAadr* mutant. Only the *pgdAadr* mutant showed a significant decrease in survival following treatment with human lysozyme. The estimated MBC_50_ (mean concentration required for 50% killing) for human lysozyme exceeded 180 µg/ml for the WT and *adr* mutant (Table 1). In contrast, the MBC_50_ was only 50 to 100 µg/ml for the *pgdA* mutant and 12.5 to 25 µg/ml for the *pgdAadr* mutant. It was concluded that there is a combined effect of peptidoglycan modifications by PgdA and Adr on resistance to human lysozyme.

**Table 1:** Sensitivity to recombinant human lysozyme.

Strain	MBC_50_ (µg/ml)
WT	>180
*adr−*	>180
*pgdA−*	50–100
*pgdA− adr−*	12.5–25

Sensitivity is shown by the estimated MBC_50_ (mean bactericidal concentration) of the wild-type (WT) strain and defined mutants. Strains tested were in a *lytA* background to eliminate effects of autolysis. Ranges were based on three independent determinations.

### Peptidoglycan modifications affect resistance to the muramidase activity of lysozyme

To test whether these effects were due to the muramidase activity of lysozyme, the enzyme was inactivated by treatment at 100°C for 60 min, which are conditions that allow lysozyme to retain its non-muramidase activity [5],[13]. Hydrolysis assays with purified peptidoglycan were used to confirm the loss of muramidase activity. The human, but not chicken egg enzyme, showed complete inactivation under these conditions (data not shown). Therefore, inactivated human lysozyme was tested for its effects on bacterial viability (Fig. 4B). The denatured enzyme lacked anti-pneumococcal activity against all of the strains tested. There was also no effect on survival when the LP9 peptide, which has been shown to confer the non-muramidase CAMP activity of lysozyme, was substituted for the intact enzyme in these viability assays at concentrations up to 200 µg/ml (data not shown). It was concluded that the combined effect of modifications by PgdA and Adr on resistance to human lysozyme is due to its muramidase, rather than its non-muramidase activity.

### Peptidoglycan modifications are required for lysozyme resistance *in vivo*

**Figure 5:**
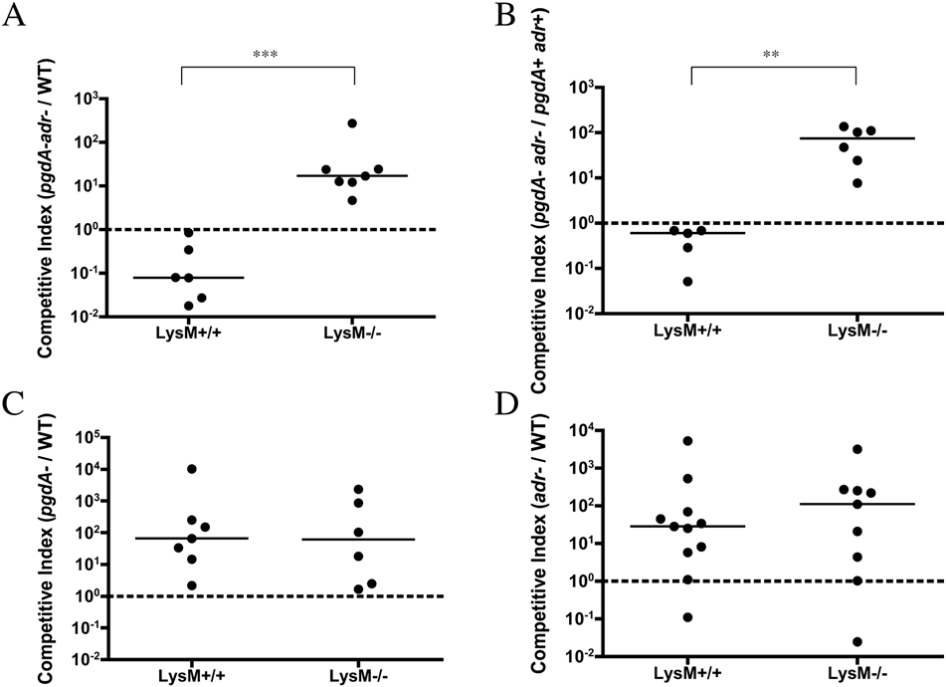
Effect of peptidoglycan modifying enzymes PgdA and Adr on relative fitness during murine colonization in the presence and absence of lysozyme M. LysM^+/+^ and LysM^−/−^ mice were challenged with equal inocula of the wild-type (WT) strain or revertant and the defined mutant indicated, and the density of each strain was determined in upper respiratory tract lavages 3 days post-inoculation. Each symbol represents the competitive index value for an individual animal. The competitive index was calculated based on the ratio of mutant to WT bacteria in nasal lavages compared to the ratio of mutant to WT bacteria in the inoculum. The dotted line is at a value of one; a value greater than one indicates the mutant out-competes the WT, a value less than or equal to one indicates the WT out-competes the mutant. A) *pgdAadr* vs. WT (^***^ p = 0.001). B) *pgdAadr* vs. the revertant (*pgdA+ adr+*) (^**^ p = 0.004). C) *pgdA* vs. WT. D) *adr* vs. WT.

The *lytA^+^* strains characterized *in vitro* were used to test the hypothesis that lysozyme limits colonization of mucosal surfaces. Both lysozyme sensitive (*pgdAadr*) and resistant (WT) pneumococci were able to colonize the upper respiratory tract of the mouse (data not shown). To examine more subtle differences in bacterial fitness, competition experiments were used to assess the relative ability of these strains to colonize the nasopharynx. Three days after intranasal inoculation with equivalent numbers of the WT strain and the *pgdAadr* mutant, the density of colonization of each strain was measured in nasal lavages to determine the competitive index. Prior to *in vivo* experiments we confirmed there was no effect of one strain on another during *in vitro* co-cultivation experiments (data not shown). As expected, the lysozyme-resistant WT strain out-competed the lysozyme-sensitive *pgdAadr* mutant in lysozyme-expressing mice (Fig. 5A). To confirm that the effect on colonization was due specifically to the mutations introduced into *pgdA* and *adr*, we generated corrected strains by transformation with WT genomic DNA followed by selection in the presence of lysozyme. Like the WT strain, the *pgdA+adr+* revertant was able to out-compete the *pgdAadr* mutant in lysozyme-expressing hosts (Fig. 5B). Together these results demonstrated that pneumococci with peptidoglycan modified by both PgdA and Adr are better able to persist during colonization.

To define the contribution of lysozyme during colonization, competition experiments were carried out in congenic LysM^−/−^ mice. In lysozyme-deficient hosts, unlike the lysozyme-sufficient hosts, the *pgdAadr* mutant prevailed over the WT or *pgdA+adr+* revertant (Fig. 5A and B). This observation demonstrated that the survival advantage conferred by these two peptidoglycan modifications requires lysozyme expression by the host. Moreover, without the expression of lysozyme, pneumococci that do not modify their peptidoglycan show a survival advantage during colonization. This suggests that the combined modifications by PgdA and Adr result in an *in vivo* fitness cost, but that this fitness cost is outweighed by their contribution to lysozyme resistance.

In contrast to the results with the *pgdAadr* mutant, single mutants in *pgdA* or *adr* out- competed the WT strain in both LysM^+/+^ and LysM^−/−^ mice (Fig. 5C and D). This suggests that individual modifications by either PgdA or Adr confer a fitness cost on the organism regardless of the presence of lysozyme. A further implication is that the combined effects of both modifications are required to confer a survival benefit – an advantage to the organism seen in lysozyme-sufficient, but not -deficient, hosts corresponding to the increased resistance of the WT strain to the muramidase activity of lysozyme.

### Epithelium is the source of upper airway lysozyme

To ascertain the source of upper airway lysozyme that effects colonization, we considered the contributions of the epithelia or the influx of neutrophils in response to pneumococci.

**Figure 6:**
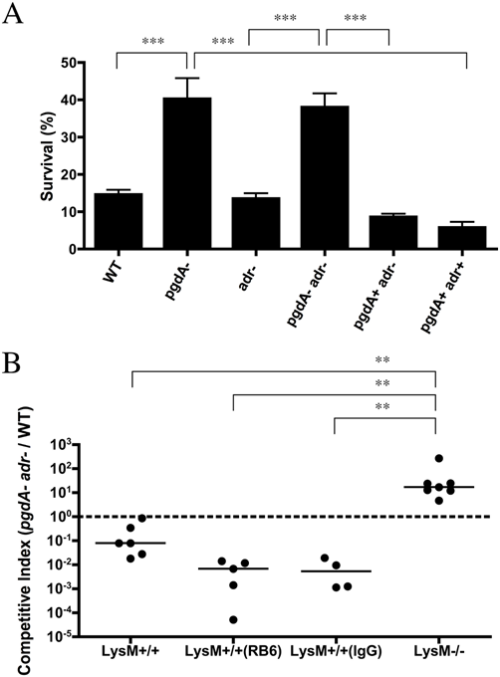
The contribution of lysozyme from neutrophils to survival and colonization of mutants lacking peptidoglycan modifications. A) Neutrophils isolated from human blood were incubated with serum opsonized bacteria and survival was assessed following a 45 min incubation. Percent survival was calculated based on viable counts (CFU/ml) relative to no neutrophil controls. (^***^ p<0.001). B) Neutrophils were depleted with anti-Ly6G antibody RB6-8C5 prior to challenge with an equal inoculum of *pgdAadr* and wild-type (WT) strains. Controls received rat IgG. Two days post-inoculation nasal lavages were obtained to quantify the competitive index. (^**^ p<0.01).

To evaluate the role of neutrophil-derived lysozyme, neutrophil-enriched peritoneal exudates were isolated from LysM^+/+^ and LysM^−/−^ mice and used in *ex vivo* killing assays. The WT strain and *pgdA*, *adr*, and *pgdAadr* mutants were equally resistant to killing by these cells whether derived from LysM^+/+^ or LysM^−/−^ mice (data not shown). Because strain TIGR4 is relatively resistant to neutrophils obtained from mice, human neutrophils were also used in killing assays (Fig. 6A). In comparison to the WT strain, mutants in *pgdA* (*pgdA* and *pgdAadr*) were significantly more resistant to neutrophil-mediated killing. Correction of the mutation in *pgdA* (*pgdA*+*adr*− and *pgdA*+*adr*+) eliminated increased resistance to killing by human neutrophils. Thus, the *pgdAadr* mutant was less resistant to lysozyme but more resistant to killing by human neutrophils, making it unlikely that the anti-pneumococcal activity of neutrophils was mediated by lysozyme. This also indicated that peptidoglycan modification by PgdA has lysozyme-independent effects on neutrophil-mediated pneumococcal killing. Additional evidence that neutrophil activity was not contributing to lysozyme-dependent differences among strains came from competition experiments in which mice were depleted of neutrophils. Administration of the monoclonal antibody RB6-8C5, which targets Ly6G-expressing cells (or rat IgG control), to LysM^+/+^ mice prior to bacterial challenge had no effect on the ability of the lysozyme-resistant WT strain to out-compete the lysozyme-sensitive *pgdAadr* mutant (Fig. 6B).

**Figure 7:**
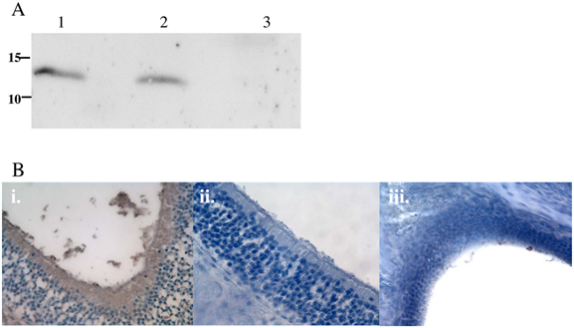
Expression of lysozyme M in the mouse nasopharyx. A) Western blot of nasal lavages from mice colonized with the wild-type strain for two days incubated with antisera to lysozyme M. Lane 1: LysM^+/+^, rat IgG control. Lane 2: LysM^+/+^, neutrophil depletion with mAb RB6-8C5. Lane 3: LysM^−/−^. Size markers are in kilodaltons. B) Immunohistochemistry on paraffin-embedded tissue sections through the nasal tissue 24 hrs post-inoculation with the wild-type strain. Staining with i) anti-lysozyme M (LysM^+/+^), ii) no primary antibody control (LysM^+/+^), and iii) anti-lysozyme M (LysM^−/−^). Sections were counterstained with hematoxylin. Magnification 400×.

The source of lysozyme in nasal lavages was also examined using an antibody to the mouse enzyme. Lysozyme M was detected in Western blots on lavage samples from LysM^+/+^ but not LysM^−/−^ colonized mice (Fig. 7A). Consistent with prior observations, lysozyme P was not detected in these blots of upper respiratory tract samples [6],[23]. There was no significant effect of neutrophil depletion on the presence of lysozyme in lavage samples from colonized mice. Using immunohistochemistry on tissue sections through the nasal passages, lysozyme was detected along the epithelial surface and mucoid material in the nasal lumen of LysM^+/+^, but not LysM^−/−^, colonized mice (Fig. 7B). It was concluded that the epitheilia rather than neutrophils was the major source of upper airway lysozyme that impacts pneumococcal colonization.

### Other effects of cell wall modifications

**Figure 8:**
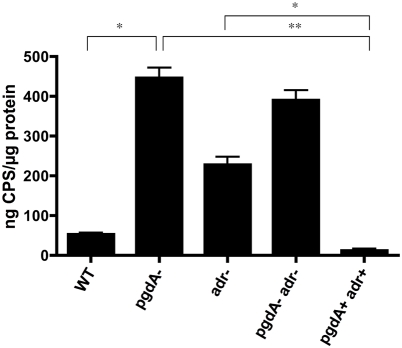
Effect of peptidoglycan modifying enzymes PgdA and Adr on expression of capsular polysaccharide (CPS). Sonicates of the bacterial strain indicated were used in a capture ELISA to measure the amount of cell-associated type 4 CPS produced relative to the total amount of protein. Values are relative to a standard with purified type 4 CPS and based on four independent determinations ±S.D. (^*^ p<0.05, ^**^ p<0.01).

Our observations showed that individual peptidoglycan modifications have lysozyme-independent effects on both bacterial fitness during colonization, and on resistance to neutrophil-mediated killing. This may be due to the effects of peptidoglycan modifications on other molecules that are attached to or associated with the pneumococcal cell wall. Specifically, an alteration in amounts of CPS, which is covalently attached to peptidoglycan [20], may have dramatic effects on fitness during colonization and resistance to neutrophil-mediated killing [24],[25]. Therefore, we assessed whether mutations in *pgdA* and *adr* change surface expression of the pneumococcal capsule. A sensitive capture ELISA was used to quantify amounts of cell-associated CPS (Fig. 8). In comparison to the WT strain or corrected mutant (*pgdA*+*adr*+), *pgdA* mutants showed significantly elevated levels of type 4 CPS. This ∼8-fold increase in immunoreactive CPS/µg total cellular protein could account for PgdA-mediated, lysozyme-independent effects on killing by neutrophils and enhanced survival during colonization by the *pgdA* mutant [26]. These findings demonstrate that, in addition to their effect on resistance to lysozyme, peptidoglycan modifications may impact major cell surface structures linked to the cell wall.

## Discussion

Our study demonstrates that host expression of lysozyme effects bacterial colonization. Specifically, we show that lysozyme is an important antibacterial factor on heavily colonized mucosal surfaces such as those lining the upper airway. These findings extend descriptions of the role of lysozyme in protecting the normally sterile lower airway [3],[8],[9]. The *in vivo* effects of the enzyme were attributed to lysozyme M, since lysozyme P was not detected in the upper respiratory tract secretions or tissue staining. As expected, the ability of bacteria to resist lysozymes' antibacterial properties dictates success at these sites. For the pneumococcus, resistance to human lysozyme *in vitro* and mouse lysozyme *in vivo* is dictated by the synergistic effects of two distinct modifications to the glycan strands of its cell wall. Even though each of these modifications alone, or in combination, diminishes bacterial fitness for colonization in the absence of lysozyme, together they provide a significant survival advantage when lysosyme is abundant.

Although two different antibacterial properties have been ascribed to lysozyme, only its enzymatic muramidase activity appeared to be relevant to pneumococcal survival. Inactivation of its muramidase activity by denaturing human lysozyme was sufficient to completely eliminate its ability to kill *S. pneumoniae*. Moreover, the cationic antimicrobial peptide (CAMP) activity of the human-derived peptide LP9 was ineffective against the pneumococcus. It remains possible that, as is the case for *S. aureus*, other cell wall features of the pneumococcus account for its resistance to the non-muramidase activity of lysozyme [13],[19]. Specifically, d-alanylation of *S. pneumoniae* cell wall-associated teichoic acids has been shown to contribute to resistance to antimicrobials, such as nicin and gallidermin [27]. This would be consistent with results in *S. aureus* where d-alanylation contributed to resistance to several antimicrobials, including lysozyme, and the peptide LP9 [13]. On the other hand, the pneumococcal strains generated in this study differed from *S. aureus* in that acetylation by Adr (OatA in *S. aureus*) by itself does not confer sufficient resistance to the lytic effects of lysozyme [18]. Our study confirmed that Adr contributes to lysozyme resistance, as had been shown previously by Crisóstomo *et al* [21], although we were only able to show an effect of the *O*-acetyltransferase Adr in conjunction with the *N*-deacetylase PgdA. This may have been due in part to differences between pneumococcal strains; Crisóstomo *et al* used an unencapsulated laboratory strain, while our studies were conducted using a clinical isolate. The extent to which these strains acetylate peptidoglycan residues could be different, and might account for the limited role of Adr in lysozyme resistance observed in this study.

Our results are also based on testing of mammalian rather than chicken egg enzyme, which may have more potent muramidase activity. This may be due in part to slightly different amino acid residues present in the catalytic cleft of each enzyme [28],[29],[30]. The amino acid sequences of chicken egg and human lysozyme are 57% identical and 76% similar by sequence comparison (blastp), whereas lysozyme M and human lysozyme are 76% identical and 86% similar. Although these differences in sequence do not lead to alterations in protein structure [28], they could affect affinity for substrates, which could lead to the observed difference in enzymatic activity.

Although many species modify their peptidoglycan by either *O*-acetylation or *N*-deacetylation, few organisms have thus far been described as having both modifications [31]. Aside from *S. pneumoniae*, these include *Bacillus cereus* and *Lactobacillus fermentum* [32],[33],[34],[35]; both *O*-acetylation and *N*-deacetylation have also been associated with lysozyme resistance, however the combined effects of both modifications in these organisms has not been determined. Our results for *S. pneumoniae* suggest that both modifications may be needed for full resistance to lysozyme. The aggregate effects of bacterial surface modifications that affect lysozyme resistance other than *O*-acetylation and *N*-deacetylation have also not been explored.

An additional observation was that peptidoglycan modifications affect characteristics of the bacterial cell apart from its resistance to lysozyme. In particular, we show that mutation of *pgdA* is sufficient to alter growth characteristic dictated by its endogenous amidase (LytA) and to markedly increase levels of cell-associated CPS. PgdA cleaves an amide bond leaving a charged amino group on glucosamine residues of the glycan backbone [10]. Mutation of *pgdA*, therefore, will result in a less positively charged cell wall, which could impact molecules attached to and associating with peptidoglycan. The efficiency of attachment of anionic type 4 CPS, which is covalently bound to the surface [20], could be affected by this difference in charge. Similarly, it would be interesting to determine if peptidoglycan modifications affect other cell surface structures such as wall teichoic acid (C-polysaccharide), which is also covalently attached to peptidoglycan, albeit at a different position [20]. An increase in cell-associated CPS, or other effects on the cell surface, could inhibit phagocytosis and explain the decreased killing of the *pgdA* mutants by human neutrophils. Altered cell surface characteristics of *pgdA* and *adr* mutants could also account for their increased fitness during colonization in the absence of lysozyme. Vollmer *et al.* previously reported that a *pgdA* mutant was less virulent in a pneumococcal model of bloodstream infection – a result that would seem to be at odds with their decreased killing by neutrophils and increased fitness on the mucosal surface [22]. Mutants in *pgdA* generated in our study, however, showed attenuated growth in human serum compared to the WT strain (data not shown). This demonstrates the potentially pleomorphic and complex effects of peptidoglycan modification on bacterial growth and survival in different environments. It would be interesting to determine whether the expression of peptidoglycan modifying enzymes is differentially regulated under conditions where the requirement for lysozyme resistance may vary.

Mucosal lysozyme may have direct effects on colonization through its ability to lyse pneumococci that do not express PgdA and Adr. Alternatively, degradation of peptidoglycan could release bacterial products recognized by the host which enhance inflammation, and indirectly stimulate bacterial clearance. These inflammatory mediators may include muramyl dipeptide (MDP), which signals through the nucleotide-binding oligomerization domain protein 2 (Nod2) cytoplasmic pathway [36],[37]. Adr could affect both sensitivity to lysozyme and signaling triggered by MDP since its MurNAc component would be modified by acetylation. In this regard, peptidoglycan modifications have been shown to effect signaling through Nod1, which recognizes the *meso*-diaminopimelic acid (*meso*-DAP) fragment of the cell wall [38],[39], in response to *Listeria monocytogenes* [16]. The relative contributions to pneumococcal clearance of the lytic versus inflammatory effects of lysozyme, and the effects of peptidoglycan modifications on the latter, have not yet been explored.

In summary, the host uses lysozyme to target a conserved feature of peptidoglycan, the glycosidic bond between MurNAc and GlcNAc. To counter this host defense mechanism, the pneumococcus modifies both of these residues to decrease hydrolysis of the bond that links them, despite a significant fitness cost associated with altering the glycan backbone of its cell wall. Our findings demonstrate that the peptidoglycan degrading activity of lysozyme exerts a substantial selective pressure on residents of mucosal surfaces.

## Materials and Methods

### Bacterial strains and growth conditions

All strains were created in a type 4 background; using a clinical isolate and genome-sequenced strain TIGR4 [40]. Pneumococci were grown in semisynthetic casein plus yeast extract (C+Y) broth, pH 6.8, at 37°C without agitation or on tryptic soy agar (TSA) plates supplemented with catalase (5,000 U/plate; Worthington, Lakewood, NJ) at 37°C in 5% CO_2_. TSA was supplemented with neomycin (5 µg/ml), spectinomycin (100 µg/ml), or kanamycin (200 µg/ml) where indicated. Strains were also co-cultivated under these conditions to ensure there were no effects of one strain on growth of the other *in vitro*. For genetic transformations, bacteria were grown from a low inoculum in C+Y at 37°C to an OD 620 nm of 0.15. Fifty-µl aliquots were added to 950 µl C+Y pH 8 with 10 ng/ml of synthetic competence-stimulating peptides 1 and 2, 10 µl of 100 mM CaCl_2_, and approximately 100 pg/ml of DNA. Reactions were incubated at 30°C for 40 minutes and then transferred to 37°C for an additional 90 minutes before being plated on selective medium.

### Generation of mutant and revertant strains

#### *pgdA* mutant strains

The 5′ end of *pgdA* was amplified using primers 1 and 2 (primer 1: 5′-TGTAGTCTGAGAAGACTTGGTAGG-3′, primer 2: 5′-ATTATTTCCTTCCTCTATTTATATCAT-3′), the 3′ end of *pgdA* was amplified using primers 3 and 4 (primer 3: 5′-GATGAATTGTTTTAGGCAAGAAAAAA-3′, primer 4: 5′-ATAAATGATAAGAATCTAAGACCGC-3′). A kanamycin resistance cassette was obtained from the Janus cassette [41] using primers 5 and 6 (primer 5: 5′-AGAGGAAGGAAATAATAAATGGCTAA-3′, primer 6: 5′-CTAAAACAATTCATCCAGTAAAATATA-3′). Primers 2 and 3 were designed with sequences complementary to primers 5 and 6 respectively, to allow for correct orientation of the kanamycin resistance cassette in between the flanking regions of *pgdA*. PCR products generated with these primers were purified using the QIAquick PCR purification kit (Qiagen Sciences, Germantown, MD) and incubated together in an overlap extension PCR. The reaction conditions were as follows: denaturation at 94°C for 2 min, 2 cycles of denaturation at 92°C for 30 sec, annealing at 40°C for 1 min, and extension at 68°C for 7 min, 33 cycles of denaturation at 92°C for 30 sec, annealing at 55°C for 1 min, extension at 68°C for 7 min, and a final extension at 68°C for 8 min. PCR products were then used in a transformation as described above with selection for kanamycin resistance. Mutant *pgdA* strains were confirmed by PCR with primers 1 and 4 followed by sequencing of the product.

#### *adr* mutant strains

The 5′ end of *adr* was amplified using primers 7 and 8 (primer 7: 5′-CAGATTCACCAATCAAATATCGTTTG-3′, primer 8: 5′-GAACGAAAATCGAATCAAGGAAAACCATTTAATGCGC-3′), the 3′ end of *adr* was amplified using primers 9 and 10 (primer 9: 5′-CCCTTGCATATTGCAGACAGCTCCAGACAAGCC-3′, primer 10: 5′-TTCGTGGCCAAGAATGGTACCAC-3′), and a spectinomycin resistance cassette from plasmid pJL74 [42] was amplified using primers 11 and 12 (primer 11: 5′-TTTTCCTTGATTCGATTTTCGTTCGTGAATAC-3′, primer 12: 5′-AGCTGTCTGCAATATGCAAGGGTTTATTGTTTTC-3′). Primers 8 and 9 were designed with sequences complementary to primers 11 and 12 respectively, to allow for correct orientation of the spectinomycin resistance cassette in between the flanking regions of *adr*. Overlap extension and transformations were performed as described above, with *adr* colonies selected by resistance to spectinomycin resistance. Mutant *adr* strains were confirmed by PCR with primers 7 and 10 followed by sequencing of the product.

#### Additional mutant strains

To generate the *pgdAadr* mutant, lysate from the *pgdA* strain was transformed into the *adr* background, and mutations were confirmed as described above. *lytA* mutants were created using lysates from *pgdA*, *adr*, and *pgdAadr* strains and transformed into a non-autolytic TIGR4 mutant with a spontaneous deletion within *lytA* [43]. *lytA* mutant strains were confirmed with primers 13 and 14 (primer 13: 5′-GCGCGGATCCCTTTTTAGTCTGGGGTG-3′, primer 14: 5′-GCGCCTGCAGATGACAAAACAAGGAA-3′).

#### Revertant strains

Revertant strains were created by transforming the *pgdAadr* mutant with WT lysate, followed by serial passage in chicken egg lysozyme (100 µg/ml) to select for resistant transformants. The *pgdA+adr−* strain has a parental *pgdA* and a mutant *adr* gene, while the *pgdA+adr+* strain is parental in both the *pgdA* and *adr* genes. Colonies were patched onto plates containing kanamycin or spectinomycin and the genotype of those that had lost resistance was confirmed by PCR as described above.

### *In vitro* lysozyme sensitivity

Bacterial growth of broth cultures was measured by optical density at an absorbance of 620 nm. Chicken egg white (Sigma, St. Louis, MO) or recombinant human lysozyme (Ventria Bioscience, Fort Collins, CO) was added to PBS at a stock concentration of 10 mg/ml. Broth cultures at mid-log phase (OD 620 nm = 0.3) were divided and received either lysozyme (100 µg/ml) or vehicle control. Where specified, lysozyme from the stock solution was inactivated by treatment at 100°C for 60 min. LP9, a synthetic human lysozyme-derived peptide (_107_R-A-W-V-A-W-R-N-R_115_) (GenScript Corporation, Piscataway, NJ), was used at a concentration of 200 µg/ml. Following addition of lysozyme, growth was monitored for 5 hrs. For viable counts an aliquot was removed and serial dilutions plated. Mean bactericidal concentrations (MBC_50_) were estimated by treating mid-log phase *lytA* mutant strains with recombinant human lysozyme. 10^2^ bacterial cells were resuspended in Hank's buffer with Ca^2+^ and Mg^2+^ (Gibco, San Diego, CA), and 0.1% gelatin (+++ solution), and were incubated with varying concentrations of human lysozyme: 180 µg/ml, 100 µg/ml, 50 µg/ml, 25 µg/ml, or 12.5 µg/ml. Reactions were incubated for 60 min at room temperature, and then plated to determine viable counts. MBC_50_ values were determined based on viability relative to reactions at time 0 min, and relative to no treatment controls.

To purify cell walls of pneumococci, cultures were first grown to exponential phase as described above. Cell walls were then purified as previously described [18]. Two-mg of crude cell walls were resuspended in 1 ml of solution (100 mM Tris Buffer pH 7.5, 10 mM CaCl_2_, and 100 µg trypsin) and incubated overnight at 37°C with agitation. Trypsin was inactivated by incubation at 65°C in 1% sodium dodecyl sulfate (SDS). Cell walls were then washed three times in distilled water to eliminate SDS and resuspended in dH_2_O to an OD 600 nm = 0.6. Hydrolysis was measured by the decline in absorbance (OD 600 nm) over 120 min in the presence of lysozyme (100 µg/ml). Percentage of hydrolysis was expressed relative to the absorbance of each sample at time 0 min.

### Murine model of bacterial competition

Six to 8-week-old female FVB/NJ mice were obtained from Jackson Laboratories, Bar Harbor, ME. Lysozyme deficient mice (LysM^−/−^) were generated in the FVB/NJ background (by T. Graf) by insertion of the Enhanced Green Fluorescent Protein into exon 1/intron 1 of the lysozyme locus. LysM^−/−^ mice were backcrossed through ten generations into the FVB/NJ background [3],[44]. Animals were housed in accordance with Institutional Animal Care and Use Committee protocols. All strains were animal passaged prior to use in experiments and stored at −80°C in 20% glycerol. Inocula consisted of a total of 10^7^ mid-log phase PBS-washed bacteria in 10 µl PBS (5×10^6^ WT or revertant cells plus 5×10^6^ mutant cells). Inocula were plated onto the appropriate selective media to confirm the concentration of each strain. Three days after intranasal inoculation, mice were sacrificed, the trachea cannulated, and 200 µl of PBS was instilled. Lavage fluid was collected from the nares and serially diluted in PBS. Total pneumococci were enumerated by plating onto media supplemented with neomycin to prevent the growth of contaminants. Selective media was used to quantify the proportion of WT or revertant vs. mutant bacteria in lavages. The lower limit of detection was 20 CFU/ml of lavage fluid.

### Phagocytic killing assays

Neutrophils were isolated from human blood by density centrifugation on a Ficoll gradient using Mono-Poly Resolving Medium according to the manufacturer's instructions (MP Biomedicals, Irvine, CA). The neutrophil-enriched fraction was collected and washed with Hank's buffer without Ca^2+^ or Mg^2+^ (Invitrogen, Carlsbad, CA) with 0.1% gelatin. Cells were counted using trypan blue staining and adjusted to a density of 7×10^6^ cells/ml in Hank's buffer with Ca^2+^ and Mg^2+^ (Gibco, San Diego, CA), and 0.1% gelatin (+++ solution). Bacterial strains were grown to mid-log phase, PBS-washed, and resuspended in +++ solution. 10^2^ bacterial cells (in 10 µl) were pre-opsonized with infant rabbit serum (40 µl) (Pel-Freez, Rogers, AR) for 30 mins at 37°C with rotation. Neutrophils were then added to reactions (10^5^ cells per reaction in 40 µl) with +++ solution (110 µl) and incubated 45 min at 37°C with rotation. Reactions were stopped by incubation at 4°C, neutrophils were lysed with dH_2_O, and viable counts of bacteria were determined. Percent survival was determined relative to control reactions lacking neutrophils.

### Neutrophil depletion

Monoclonal antibody (mAb) RB6-8C5, a rat anti-mouse IgG2b directed against Ly-6G on the surface of murine myeloid (and limited subpopulations of lymphoid) lineage cells, was purified from ascites of nude mice given the RB6-8C5 hybridoma [45],[46]. To deplete neutrophils, 145 µg of mAb/animal was given by intraperitoneal (i.p.) injection 24 hours prior to intranasal challenge with bacteria. This dose was previously shown to result in peripheral blood neutropenia (<50 granulocytes/µl) for a period of at least 48 hours [47]. Controls were given the equivalent i.p. dose of total rat IgG (Sigma, St. Louis, MO).

### Western blot analysis

Mice were colonized 48 hrs with WT *S. pneumoniae*, sacrificed, and nasal lavages obtained as described above. Nasal lavage fluid was separated by SDS-polyacrylamide gel electrophoresis on a 15% Tris-HCl gel (Bio-Rad, Hercules, CA), and proteins were transferred to a polyvinylidene difluoride transfer membrane (Thermo Scientific, Rockford, IL). Murine lysozyme was detected using a rabbit anti-mouse polyclonal IgG antibody to recombinant lysozyme M [6] and detected with enhanced chemiluminescent (ECL) anti-rabbit IgG horseradish peroxidase-conjugated secondary antibody from donkey (GE Healthcare, Little Chalfont, Buckinghamshire, UK). Secondary binding was detected with ECL Western Blotting Substrate (Pierce Biotechnology, Rockford, IL).

### Immunohistochemistry

Mice were colonized 24 hrs with WT *S. pneumoniae*, a time point prior to neutrophil influx into the nasopharynx. Mice were then sacrificed, decapitated, and heads were fixed in 4% paraformaldehyde in PBS for 48 hrs. Heads were then decalcified for 24 hrs in Cal-EX decalcifying solution (Fisher Scientific, Fair Lawn, NJ). Paraffin-embedded tissue was sectioned and rehydrated through a series of xylene and ethanol washes with decreasing concentrations of ethanol. Sections were post-fixed in 1∶1 methanol-acetone at −20°C for 10 min followed by washing in distilled water (dH_2_0). Endogenous peroxidases were blocked with 30% hydrogen peroxide in dH_2_0 for 15 min. Sections were also blocked with avidin and biotin, each for 15 mins, followed by a 10 min incubation in protein blocking reagent (Coulter/Immunotech, Miami, FL). Murine lysozyme was detected using a rabbit anti-mouse polyclonal IgG antibody to recombinant lysozyme M [6] at a 1∶500 dilution in PBT (1× PBS, 0.1% bovine serum albumin, 0.2% Triton X-100), or PBT alone for no primary antibody controls, and incubated 60 min at 37°C. Primary antibody was detected using a biotinylated goat anti-rabbit antibody (Vector Laboratories, Burlingame, CA) with a 1∶200 dilution in PBT and incubated 30 min at 37°C, followed by 30 min incubation at 37°C with horseradish peroxidase-conjugated ABC reagent (Vector Laboratories). Sections were washed with PBS between all steps. The signal was developed using a diaminobenzidine tetrahydrochloride (DAB) kit (Vector Laboratories) and developing was stopped with dH_2_0. Sections were then counterstained with hematoxylin, dehydrated in ethanol, cleared in xylene, and mounted in Cytoseal (Richard-Allen Scientific, Kalamazoo, MI).

### ELISA for capsular polysaccharide (CPS)

Cultures of strains of the same colony opacity were grown to stationary phase in 10 ml of C+Y, pelleted, and resuspended in 10 ml of PBS. Cells were adjusted to equal optical density, resuspended in 1 ml of PBS, and lysed by sonication. Capture ELISAs were then used to quantify capsule as previously described [24]. Type 4 typing sera (Statens Seruminstitut, Copenhagen, DK) was fixed to a 96-well polystyrene microtiter plate by incubating 16 hours at room temperature (RT) using a 1∶5000 dilution in 0.05 M sodium carbonate. Plates were washed five times with wash buffer (1× PBS with 0.05% Brij). Sonicate samples were added at a 1∶500 dilution and plates were incubated 2 hrs at RT with agitation, followed by five washes. A mAb to type 4 CPS was added at 1∶400 dilution in wash buffer. Goat anti–mouse IgG alkaline phosphatase conjugate was used as the secondary antibody at a dilution of 1∶10,000. Both antibodies were incubated in plates for 2 hrs at RT with agitation followed by five washes. Plates were developed using alkaline phosphatase substrate (Sigma, St. Louis, MO) in diethanolamine buffer, and were read at an absorbance of 415 nm. Total protein in each sonicate was measured using a Micro-BCA protein assay kit (Pierce Biotechnology, Rockford, IL), following the manufacturer's protocol. Purified type 4 CPS (American Type Culture Collection, Manassas, VA) was used as a standard.

### Statistical analysis

Statistical comparisons of lysozyme sensitivity, phagocytic killing assays, and amounts of CPS were calculated using a 1-way ANOVA (Kruskall-Wallis test, non-parametric) with Dunn's post-test (Prism 4, GraphPad Software, San Diego, CA). Statistical comparisions between colonization groups were computed using the Mann-Whitney test (non-parametric, two-tailed test) (Prism 4, GraphPad Software, San Diego, CA).
